# Fundamental research on the label-free detection of protein adsorption using near-infrared light-responsive plasmonic metal nanoshell arrays with controlled nanogap

**DOI:** 10.1186/1556-276X-8-274

**Published:** 2013-06-07

**Authors:** Shuhei Uchida, Nobuyuki Zettsu, Katsuyoshi Endo, Kazuya Yamamura

**Affiliations:** 1 Research Center for Ultra-Precision Science and Technology, Osaka University, 2-1 Yamada-oka, Suita, Osaka 565-0871, Japan; 2 Green Mobility Collaborative Research Center, Nagoya University, Furo-cho, Chikusa-ku, Nagoya, Aichi 464-8603, Japan

**Keywords:** Nanoparticle, Self-assembly, Localized surface plasmon resonance (LSPR), Biosensing

## Abstract

In this work, we focused on the label-free detection of simple protein binding using near-infrared light-responsive plasmonic nanoshell arrays with a controlled interparticle distance. The nanoshell arrays were fabricated by a combination of colloidal self-assembly and subsequent isotropic helium plasma etching under atmospheric pressure. The diameter, interparticle distance, and shape of nanoshells can be tuned with nanometric accuracy by changing the experimental conditions. The Au, Ag, and Cu nanoshell arrays, having a 240-nm diameter (inner, 200-nm polystyrene (PS) core; outer, 20-nm metal shell) and an 80-nm gap distance, exhibited a well-defined localized surface plasmon resonance (LSPR) peak at the near-infrared region. PS@Au nanoshell arrays showed a 55-nm red shift of the maximum LSPR wavelength of 885 nm after being exposed to a solution of bovine serum albumin (BSA) proteins for 18 h. On the other hand, in the case of Cu nanoshell arrays before/after incubation to the BSA solution, we found a 30-nm peak shifting. We could evaluate the difference in LSPR sensing performance by changing the metal materials.

## Background

In the last 10 years, we have witnessed a rapid growth in the development of highly selective and sensitive optical biosensors for the medical diagnosis and monitoring of diseases, drug discovery, and the detection of biological agents. Among the many advantages of optical biosensors, sensitivity and simple detection systems allow them to be applied widely. Optical sensing techniques are based on various sensing transduction mechanisms, fluorescence, light absorption and scattering, Raman scattering, and surface plasmon resonance (SPR) [1-3]. Especially, sensing systems using localized SPR (LSPR) have received significant research attention in recent years as a result of their potential for use as highly sensitive, simple, and label-free bio/chemical binding detection devices [4-6].

In the emergence of plasmonics, there has been much research interest in various kinds of metal nanostructures, in which confined free electrons are forced to oscillate by an incident light; the resulting collective oscillation of electrons can exhibit strong local field enhancement at a particular frequency. In the LSPR, the incoming light is absorbed or scattered by the nanostructures, and concurrently, there is an electromagnetic field enhancement close to the nanostructures. It is well established that the peak extinction wavelength, *λ*_max_, of the LSPR spectrum is dependent upon the size, shape, spacing, and dielectric properties of materials and the local environment [7-9]. LSPR has been explored in a range of nanostructure shapes such as spheres, triangles, or cubes. Major efforts have gone into studying the sensitivity of such structures to changes in the local environments and refractive index. The potential for their use as ultrasensitive detectors comes from both their high sensitivity and the short range of the associated optical fields. Therefore, this property opens a route to the sensing of local biomolecular recognition events where adsorbate-induced changes in the local dielectric environment around the nanostructures are utilized.

There is a significant demand for the development of simple, robust, and accurate optical biosensors for deployment in a wide range of applications such as the analysis of molecular structures or the detection of disease agents. Considering the use of LSPR sensing systems in the medical front, it is not satisfied only by evaluating sensitivities to the changing of the bulk refractive index or surface environment. It is noted that the detection of chemical systems including those targeting and proving molecules have to be done by LSPR sensing for practical purposes. For simple research on the present LSPR biosensor study on immunoassay, we focused on bovine serum albumin (BSA) binding onto the surface of metal nanostructures.

Such bioapplications with good performances require an excitation within 800 to 1,100 nm (the so-called optical window) to provide a deeper tissue penetration of photons with reduced photodamage effects. Several authors have taken advantage of the high permeability of the human skin and tissue to near-infrared (NIR) radiation to develop diagnostic detection tec-hniques. The use of NIR light is a promising approach for biomedical detection based on LSPR. Thus, metal nanoparticles with various shapes have been proposed to respond to NIR light. In shell-type geometries such as nanoshells and nanorings [10], interactions among electrons bound to the inner and outer surfaces of the shell give rise to the so-called plasmon hybridization [11-13], resulting in a wide range of tenability and higher sensitivities for sensing. It is well known that NIR light provides LSPR in nanoshells as the simplest nanostructure. Since sensing systems using NIR light, however, are required to improve their detection sensitivity, it is necessary to arrange as many nanostructures as possible as sensing units on the substrate.

From the abovementioned background, we propose nanoshell arrays for LSPR sensing platforms combining the best features of NIR light response. In recent years, there exist a lot of reports on various metals generating LSPR, while few researchers describe a systematic comparison to optimize sensing performance by changing the materials. In this study, we use Au, Ag, and Cu, typical materials for the plasmonic research field, for metal nanoshell arrays and experimentally and quantitatively demonstrate a suitable metal for LSPR sensing.

## Methods

### Fabrication of PS@Au nanoshell arrays

**Figure 1:**
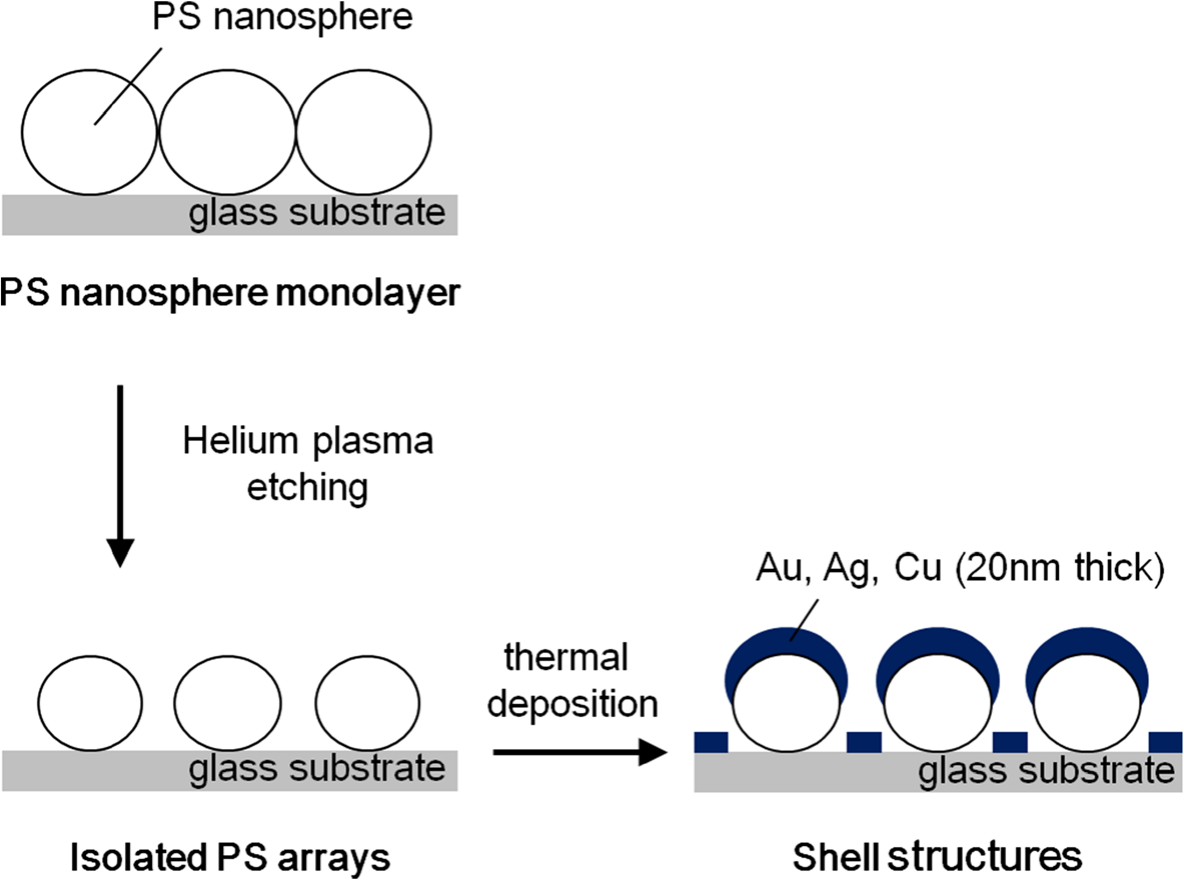
Illustration of the fabrication process of metal nanoshell arrays on substrates.

Nanosphere lithography was performed to fabricate near-infrared light-responsive plasmonic nanoshell arrays. A schematic illustration of the fabrication process is shown in Figure 1. The detailed description has been reported in our previous papers [14]. We prepared a monolayer of polystyrene (PS) nanosphere with a hexagonally close-packed structure by convective self-assembly.

The colloidal dispersion of monodispersed PS nanospheres with a mean diameter of 320 nm was purchased from Thermo Scientific Corporation (Waltham, MA, USA). The surface of PS was functionalized with a carboxylic or sulfonic functional group, which showed a *ζ*-potential of around −30 to −40 mV in pure water. The cleaned glass substrate with dimensions of 30 × 60 mm^2^ was coated with a PS thin film as an adhesion layer by spin coating. Prior to the deposition of PS nanospheres, the PS film surface was treated with helium (He) plasma under atmospheric pressure, forming a hydrophilic surface. After subsequent He plasma etching to shrink and isolate the nanospheres, we prepared metal nanostructures through a direct thermal deposition technique. We chose Au, Ag, and Cu as shell materials.

The optical properties and sensing characteristics were studied by unpolarized UV–vis-NIR extinction measurements with standard transmission geometry. The probe diameter was approximately 10 × 5 mm^2^ (HITACHI U-4000 with a CCD detector, Hitachi, Ltd., Chiyoda-ku, Japan).

### Surface functionalization of metal nanoshell arrays

We have focused on the detection of BSA binding for fundamental research to realize a label-free, sensitive, and effective immunoassay. For the investigation of BSA binding onto the surface of Au nanoshell particles, the LSPR spectrum of a nanoshell sample was firstly measured. After surface UV cleaning for 20 min, the sample was incubated with BSA in PBS buffer at the condition of 1.5 × 10^−6^ M for 18 h at room temperature. The sample was rinsed with water and nitrogen-dried, and optical properties were measured.

## Results and discussion

**Figure 2:**
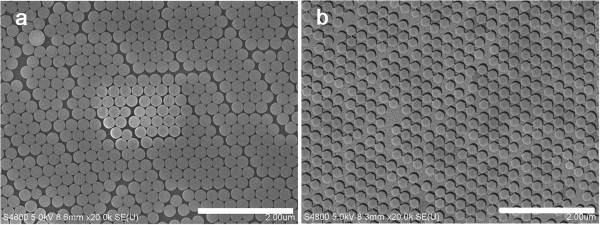
**SEM images of the (a) PS nanoparticle monolayer and (b) 240-nm Au nanoshell arrays.** The scale bars in (**a**) and (**b**) are 2 μm.

The scanning electron microscopy (SEM) image of the PS nanoparticle monolayer fabricated on glass substrates is shown in Figure 2a. In Figure 2b, presenting Au nanoshell arrays, even though structural defects coming from its initial structure were observed, the nanoshell has a monodispersive size distribution compared to other methods of achieving isolated nanoparticle arrays [15,16]. We can precisely control the diameter of nanoparticles and the gap distance by changing the plasma etching time. In this study, we arranged the interparticle distance at 80 nm for the reason that it is essential to keep substantial spacing to attach the BSA protein molecule on the surface of nanoshells.

**Figure 3:**
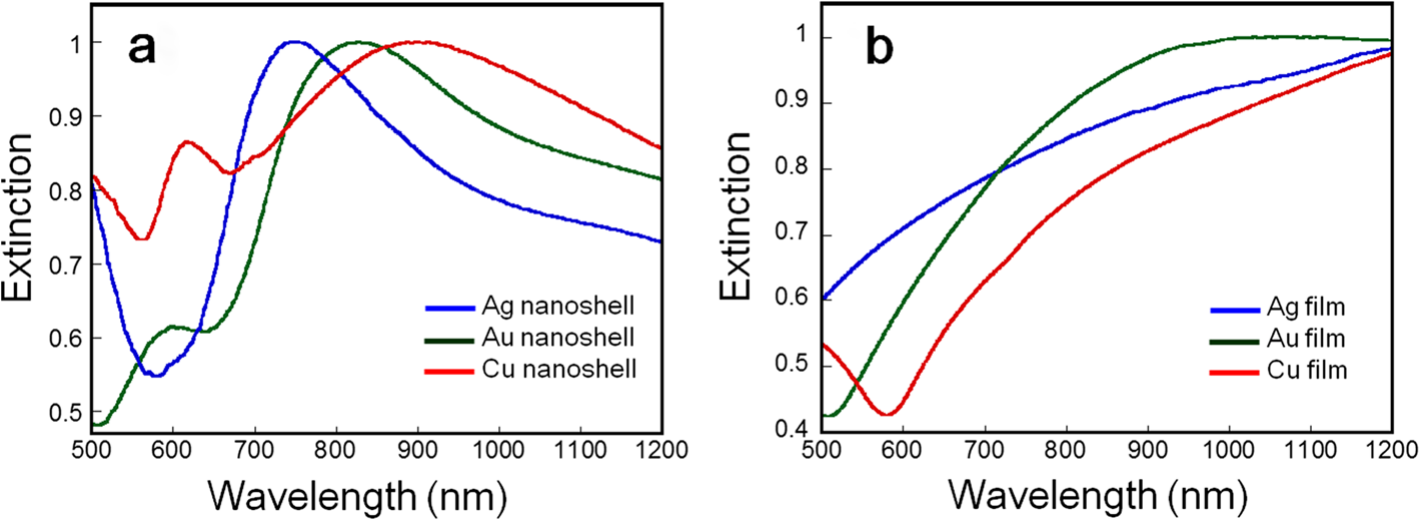
**Normalized LSPR spectra of (a) nanoshell arrays and (b) metal films on glass substrates.** Shell thickness was controlled to 20 nm. All spectra were collected in the air.

Figure 3a illustrates the normalized extinction spectra of Au, Ag, and Cu nanoshell arrays of similar size and geometry with 200 nm of core diameter and 20 nm of shell thickness. Each LSPR peak has a well-defined shape, and in the case of Au and Cu, it shows a broad shoulder around 600 nm originating from the interband transitions of bulk materials. Therefore, the interband transitions do not significantly affect the LSPR properties of Au and Cu nanoshell arrays. The LSPR *λ*_max_ of Au, Ag, and Cu were measured to be 830, 744, and 914 nm, respectively, and the full width at half maximum of the LSPR were *ca.* 300, 280, and 390 nm, respectively. These peaks were not so sharp compared to expected results in nanoshells. This is because the fabricated samples consist of nanoshell particles and a glass substrate with a metal thin film exhibiting high extinction in the NIR region as shown in Figure 3b. We anticipate that without the metal film on the glass substrate, a sharper optical peak in the NIR region can be achieved with selectively laminated metal nanoshells fabricated by plating techniques. The LSPR *λ*_max_ of Au and Cu are at longer wavelengths than that of Ag nanoshell arrays of similar structural parameters. In other research, the trend was revealed from the discrete dipole approximation method where the LSPR *λ*_max_ of Au > Cu > Ag for nanostructures of the same geometry [17]. Also, it was described that the LSPR peak of Cu nanostructures significantly red-shifted and broadened as the thickness of the oxide layer increased. In fact, our Cu nanoshell arrays included an oxide layer, and LSPR peaks might shift from their primary position. The discrepancy of the Cu LSPR *λ*_max_ between experiment and theory can be attributed to the difficulty in quantitative and ultratrace measurement. From the comparison of the LSPR of Au, Ag, and Cu nanoshell arrays with the objective of application to biosensing devices using NIR light, we conclude that Au nanoshell arrays display suitable properties that are comparable to those of Ag and Cu.

**Figure 4:**
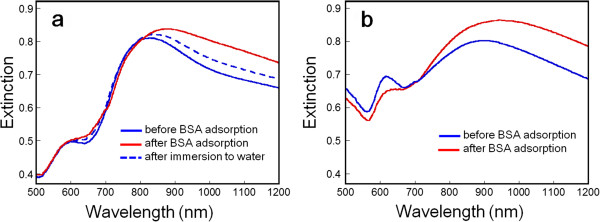
**LSPR spectra of nanoshells before/after BSA attachment in (a) Au and (b) Cu nanoshell arrays.** All spectra were collected in the air.

We have fundamentally investigated Au nanoshells on glass substrates as potential label-free optical transduction elements in a nanoscale biosensor. In this experiment, the initial extinction properties of nanoshells are measured after UV-O_3_ surface cleaning for 20 min. The detection of BSA molecule adsorption was demonstrated as shown in Figure 4a. First, the LSPR *λ*_max_ of bare Au nanoshells was measured to be 830 nm. The LSPR *λ*_max_ after incubation to the BSA solution was measured to be 885 nm, corresponding to an additional 55-nm red shift, which was a wavelength shift two times larger than that of the reported nanohole substrate as a femtomole-level LSPR sensor [18]. Also, we confirmed that this peak position was not shifted after immersion in water. Furthermore, since the BSA molecule has no selective adsorption, this peak shift was attributed to the LSPR response to the changing of the local refractive index with the adsorption of BSA, which physically adsorbed to the gold surface of nanoshells and the substrate at the gap of nanoshells. It is indicated that we can improve the detection efficiency by localizing the adsorption area of the target molecule without gold film directly laminated on the glass substrate. After immersion in water for 24 h, it is found that the *λ*_max_ of nanoshell arrays returned to 834 nm. It is revealed that the red shift of peak position was due to the physical adsorption of BSA proteins. Additionally, it is indicated that the LSPR peak did not return to its initial position because of the incomplete removal of BSA only with immersion in water. For application to bio/chemical detection devices, it should be noted that the signal transduction mechanism in this nanosensor is a reliably measured wavelength shift in the NIR region.

Figure 4b shows the change of LSPR properties taken from Cu nanoshell arrays before/after incubation to the BSA solution. In the air, the LSPR *λ*_max_ of the bare Cu nanoshell arrays was measured to be 914 nm. Exposure to the BSA solution resulted in LSPR *λ*_max_ = 944 nm, corresponding to an additional 30-nm red shift. In the case of Cu nanoshells, they exhibited a not so low sensitivity to the adsorption of molecule relative to Au. While Cu nanoshell arrays have problems to solve about their oxide layer and chemical stability, it is possible for inexpensive Cu to substitute for Au because of its sensitivity to the adsorption of biomolecule. We could evaluate the difference in LSPR sensing performance by changing the metal materials in the experiment.

## Conclusion

In summary, we successfully fabricated uniform metal nanoshell arrays in a large area (30 × 60 mm^2^) on glass substrates and characterized the geometry and the optical properties based on the LSPR of the Au, Ag, and Cu nanoshell arrays. The LSPR *λ*_max_ of Au and Cu were at longer wavelengths than that of Ag nanoshell arrays of similar structural parameters. This result indicates that Au and Cu are superior to Ag as materials for NIR light-responsive plasmonic sensors. It is difficult to detect an ultralow amount of target molecule quantitatively and precisely because the optical properties of the Cu nanoshell arrays are significantly affected by the presence of the oxide layer. The tests on BSA binding onto the Au shell surface demonstrated a wavelength shift two times larger than that of the reported nanohole substrate as a femtomole-level LSPR sensor. Our fabrication technique and the optical properties of the arrays will provide useful information for developing NIR light-responsive plasmonic applications.

## Competing interests

The authors declare that they have no competing interests.

## Authors’ contributions

SU fabricated the metal nanoshell arrays on the substrates, measured the optical properties, carried out the BSA binding experiment, and drafted the manuscript. NZ participated in the design of the study and helped draft the manuscript. KE and KY conceived of the study, participated in its design and coordination, and helped draft the manuscript. All authors read and approved the final manuscript.
